# Dysregulation of Adenosinergic Signaling in Systemic and Organ-Specific Autoimmunity

**DOI:** 10.3390/ijms20030528

**Published:** 2019-01-27

**Authors:** Marta Vuerich, Rasika P. Harshe, Simon C. Robson, Maria Serena Longhi

**Affiliations:** 1 Department of Anesthesia, Beth Israel Deaconess Medical Center, Harvard Medical School, 330 Brookline Avenue, Boston, MA 02215, USA; mvuerich@bidmc.harvard.edu (M.V.); rharshe@bidmc.harvard.edu (R.P.H.); srobson@bidmc.harvard.edu (S.C.R.); 2 Division of Gastroenterology, Department of Medicine, Beth Israel Deaconess Medical Center, Harvard Medical School, 330 Brookline Avenue, Boston, MA 02215, USA

**Keywords:** ectonucleotidase, adenosine, adenosine receptor, autoimmunity, T-cell

## Abstract

Exact causes for autoimmune diseases remain unclear and no cures are available. Breakdown of immunotolerance could set the stage for unfettered immune responses that target self-antigens. Impaired regulatory immune mechanisms could have permissive roles in autoreactivity. Abnormal regulatory immune cell function, therefore, might be a major determinant of the pathogenesis of autoimmune disease. All current treatments are associated with some level of clinical toxicity. Treatment to specifically target dysregulated immunity in these diseases would be a great advance. Extracellular adenosine is a signaling mediator that suppresses inflammation through activation of P1 receptors, most active under pathological conditions. Mounting evidence has linked alterations in the generation of adenosine from extracellular nucleotides by ectonucleotidases, and associated perturbations in purinergic signaling, to the immunological disruption and loss of immunotolerance in autoimmunity. Targeted modulation of the purinergic signaling by either targeting ectonucleotidases or modulating P1 purinergic receptors could therefore restore the balance between autoreactive immune responses; and thereby allow reestablishment of immunotolerance. We review the roles of CD39 and CD73 ectoenzymes in inflammatory states and with the dysregulation of P1 receptor signaling in systemic and organ-specific autoimmunity. Correction of such perturbations could be exploited in potential therapeutic applications.

## 1. Introduction

Purinergic signaling relies on extracellular ATP (eATP; and other nucleoside tri- and di-phosphates) and the main product of their hydrolysis, viz. adenosine, to modulate adaptive and innate immune responses. Release of nucleotides in the extracellular environment following tissue injury might result initially in the activation of P2 receptors on target cells, which is then rapidly followed by the hydrolysis of these nucleotides into adenosine by the tandem functions of select ectonucleotidases [1]. P2 receptors are virtually expressed on all immune cells and are mainly associated with pro-inflammatory responses [2]. On the other hand, extracellular adenosine signaling is suppressive and is mediated upon engagement of P1 receptors. These consist of four G-protein-coupled receptors, namely A1, A2A, A2B, and A3 receptors (A1R, A2AR, A2BR, A3R). Of these, A2AR and A2BR are mainly endowed with immunoregulatory functions [3,4].

In this review, we will discuss the role of ENTPD1/CD39 and CD73 ectoenzymes and focus on the effects of extracellular adenosine on P1 receptors, in the context of systemic and organ-specific autoimmune conditions.

Ectonucleotidases are expressed on the plasma membrane of immune cells and belong to several enzymatic families, which have been structurally and functionally characterized [5,6]. ENTPD1/CD39, the prototype member of the NTPDase family, is a rate-limiting ectoenzyme that hydrolyzes ATP into AMP, which is then further degraded into adenosine by the ecto-5′-nucleotidase/CD73 [5]. Once generated, adenosine activates P1 receptors on target cells; it can undergo catalysis into inosine by adenosine deaminase; or, alternatively, this nucleoside can be recaptured via cellular re-uptake and used for purine salvage pathways [5]. ENTPD1/CD39 and CD73 are constitutively expressed in human innate and adaptive immune cells such as monocytes, granulocytes, B-cells and T-cell subsets, including regulatory and memory subpopulations. Pathological conditions, inflammatory stress and exposure to specific compounds (e.g., aryl-hydrocarbon-receptor [AhR] ligands) markedly impact ectonucleotidase expression and functionality [6,7,8,9].

Both ENTPD1/CD39 and CD73 have been identified as major contributors to murine and human regulatory T-cell (Treg) function [10,11,12,13]. Adenosine signaling stabilizes Foxp3 expression [14] while the combined CD39/CD73 activity protects from the inhibitory and pro-apoptotic effects of P2X7R signaling through eATP scavenging [15]. Notably, adenosine not only inhibits effector T-cells via A2AR activation [16], but also increases ENTPD1/CD39 levels in an autocrine manner by acting on this same A2AR on regulatory cells [17,18]. A2AR signaling on Foxp3^+^ Tregs induces CTLA-4 and PD-1 expression, enhances cell proliferation and promotes Treg/dendritic cell (DC) interactions via Epac1-Rap1-dependent pathways [19].

Furthermore, recent investigations have revealed a competitive effect of A2AR expressed by γδ T-cells on adenosine-mediated Treg functions. Once activated, γδT-cells markedly upregulate adenosine receptors, depriving Foxp3^+^ T-cells of local adenosine, thus inhibiting their expansion [20,21]. In a comparable fashion, A2BR mRNA is upregulated on activated regulatory cells and endotoxin-induced Treg accumulation is impaired in A2BR deficient mice [22].

ENTPD1/CD39 activity also supports the differentiation and function of IL-27-induced Tr-1 cells and ENTPD1/CD39 genetic deletion results in impaired differentiation, due to increased P2X7R response that limits AhR signaling [23]. Interestingly, Tr-1 cells do not express CD73 and, in this setting, adenosine generation relies on the combined activity of ENTPD1/CD39 on Tr-1 cells and on CD73 present on adjacent DCs or effector cells [23]. IL-6 and TGF-β induce ENTPD1/CD39 and CD73 expression in pro-inflammatory Th17-cells, conferring a non-pathogenic immunoregulatory phenotype [24]. These so-called suppressor Th17 (SupTh17) cells further upregulate Foxp3 and ENTPD1/CD39 expression in response to AhR agonists [7]. The adenosine generated stabilizes SupTh17-cell differentiation likely by promoting the expression of stem cell-related transcription factors (tcf-7 and lef-1) that limit differentiation into Th1-like phenotypes [25].

ENTPD1/CD39 has been also detected in long-lived memory T-cells and exhausted effector T-cells [26,27] where it impacts survival through the regulation of the mammalian-target-of-rapamycin (mTOR) [28].

In human peripheral blood mononuclear cells (PBMCs), exposure to TNF-α increases the percentage of CD73^+^CD4^+^ T-cells [29]. CD73 expression on conventional CD8^+^ and CD4^+^ T-lymphocytes can be also induced by the active form of vitamin D, retinoic acid and TFG-β [30,31]. On the other hand, terminally differentiated CD8^+^ T-cells potently decrease levels of CD73 expression [25]. A2AR activation in naïve/memory CD8^+^ T-cells regulates the transition to effectors by inhibiting Wnt signaling [25,26]; whereas, in activated effector T-cells, A2AR stimulation induces cAMP and decreases pro-inflammatory cytokine (e.g., TNF-α, IL-6) release [32]. Accordingly, global A2AR deletion or blockade results in uncontrolled inflammatory responses with serious tissue damage resulting from the dysregulated immune response [32].

T-cell responses to adenosine can be inhibited by G protein-coupled receptor kinase (GRKs)-mediated phosphorylation of P1 receptors or by PKA-mediated desensitization, consequent to exposure to adenosine. Interference with P1 receptor response signaling can also derive from upregulation of immune cell phosphodiesterases (PDE) or proteins that limit the A2A C-terminal-cAMP signaling [33,34,35]; and may also depend on the presence of A2AR splice variants that abrogate the Ac-dependent cAMP responses [36].

## 2. Systemic Autoimmune Disorders

### 2.1. Systemic Lupus Erythematosus

Systemic lupus erythematosus (SLE) is a chronic systemic autoimmune disorder leading to multi-organ inflammation. SLE may present with a wide spectrum of clinical manifestations and is frequently characterized by recurring episodes of relapse and remission.

**Figure 1:**
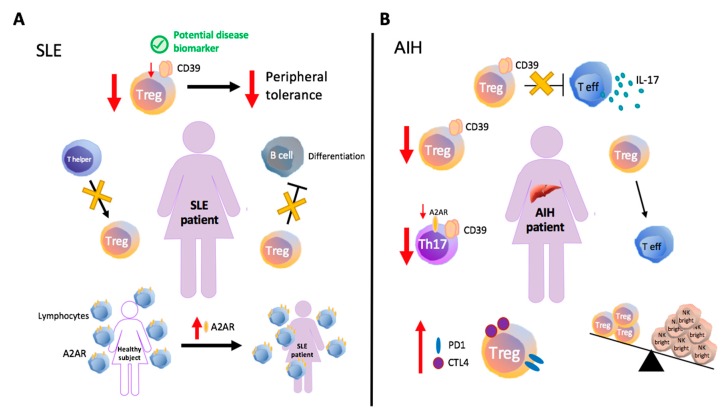
Immune and purinergic dysregulation in systemic lupus erythematosus (SLE) and autoimmune hepatitis (AIH). (**A**) In SLE, defects in ENTPD1/CD39 expression and impaired Treg function have been associated with loss of peripheral tolerance. Studies conducted in active SLE patients have indicated impaired suppression of B-cell differentiation and identified the abnormal generation of regulatory T-cells (Treg) as result of limited transition from inducer/helper to suppressor phenotype. Upregulation of A2AR is detectable in SLE patients, likely being linked to activation of compensatory pathways. (**B**) In type 1 autoimmune hepatitis (AIH-1), CD39^+^ Treg cells display impaired suppression of IL-17 production by CD4^+^ effectors. Acute AIH patients present a low ratio between Tregs and NK bright cells, a specific NK subset with activated effector phenotype. Activated memory phenotype and signs of exhaustion, including increased CTLA-4 and PD-1 levels are also typical of AIH Tregs. Reduced CD39^+^ Treg and CD39^+^ Th17-cell frequencies positively correlate with the disease progression and might result from cell instability upon pro-inflammatory challenge, with increased rate of conversion into effector lymphocytes. The reduction in CD39^+^ Th17-cell numbers, also associates with lower A2AR expression (see text below).

There is increasing evidence supporting the role of adenosine as a protective mediator, in SLE; adenosine is operational via adenosine receptors (Figure 1A). In a murine model of lupus nephritis, treatment with A2AR agonists results in significant improvement of renal histopathology, this being associated with decreased blood urea, creatinine and proteinuria [37]. ENTPD1/CD39^−/−^ and CD73^−/−^ mice show more pronounced endothelial cell dysfunction and exaggerated neutrophil extracellular “trap” release in response to intraperitoneal administration of pristane, in a model of lupus, when compared to wild type (WT) controls.

Further studies have shown that CD73^−/−^ mice have more activated B-cells in the spleen and higher levels of plasma cell-free DNA, whereas ENTPD1/CD39 deficiency results in greater Th17-cell expansion [38].

Accordingly, in SLE patients, loss of immunotolerance has been linked to defects in ENTPD1/CD39 expression and impaired Treg function, suggesting that ENTPD1/CD39 deficient Tregs could be associated with disease or might serve as disease biomarkers [39]. A study conducted in active SLE patients has revealed abnormal generation of suppressor T-cells as result of the limited transition from inducer/helper to suppressor phenotype. The dysfunction was observed in both spontaneous and adenosine-inducible suppressor cells and, interestingly, this abnormality corrected upon disease remission [40]. Another line of investigation has correlated immune dysregulation in SLE with resistance of T-cells to adenosine-mediated effects [40].

To this end, SLE-derived T-cells lack adenosine receptor-coupled adenylate cyclase activity, possibly contributing to impaired immunoregulation. T-lymphocytes from both SLE patients and healthy subjects express A2R, but not A1R [41]. Although no differences have initially been observed in A2R density and responses in SLE, a recent study has shown upregulation of A2AR in these patients [41,42]. This finding might be linked with the activation of compensatory pathways, given that A2AR activation is a strong immunoregulatory signal. Indeed, A2AR levels inversely correlate with disease activity and the use of A2AR agonists might represent a potential therapeutic approach to correct immunoregulation in SLE [42].

### 2.2. Rheumatoid Arthritis

Rheumatoid arthritis (RA), as in the case of SLE, predominantly impacts females and is characterized by joint inflammation and synovial tissue hyperplasia. These lesions eventually result in cartilage and bone damage with increasing deformity and disability. Recent discoveries have resulted in novel and improved therapies that, however, remain non curative [43].

One first-line treatment choice for RA involves the use of methotrexate (MTX), the anti-inflammatory action of which has been linked to increased levels of adenosine. This increased engagement of adenosine via P1 receptors activates intracellular cascades, thereby promoting an overall anti-inflammatory state.

Interestingly, studies conducted in murine models of arthritis have shown that CD39 blockade and decreased adenosine generation reverse the therapeutic effect of MTX, while non-responder patients express lower pre-treatment levels of CD39/ENTPD1 [44]. Low CD39 density on Treg and MTX resistance have been both associated with alterations in TGFβRII and CREB1, which are TGF-β signaling factors, in turn leading to CD39/ENTPD1 expression. In this regard, lower expression of TGFβRII and CREB1 or decreased levels of p-SMAD2 and p-CREB might both result in MTX resistance [45].

Notably, aspects of adenosine signaling protect from MTX toxicity. Five SNPs within the A2AR gene have been linked to increased MTX gastrointestinal toxicity, serving as useful markers for high risk patients prior to treatment [46].

Adenosine signaling is also involved in the protective effects of fructose 1,6 bisphosphate (FBP). FBP administration attenuates experimental arthritis promoting immunoregulatory pathways mediated by CD39/CD73 and A2AR signaling [47]. In collagen induced arthritis, IL-6 release by pro-inflammatory cells negatively impacts the frequency of CD39^+^ Tregs in lymph nodes and spleen, this effect being abrogated by antibody-mediated IL-6 neutralization [48]. Similarly, TNF-α accumulation causes de-phosphorylation of Foxp3 leading to Treg functional impairment [49]. As a compensatory response, RA patients present higher Treg frequencies in the joints, associated with increased CD39 function and lower adenosine deaminase activity [50]. These synovial Foxp3^+^CD39^+^CD25^+^ T-cells, however, while effectively suppressing IFNγ, TNF-α and IL-17F, fail to control IL-17A secretion by effector T-cells [51].

In addition to CD39, CD73 also plays a protective role in RA. In this context, patient-derived Foxp3^+^ cells obtained from the synovium display low levels of CD73 [51]. Further, CD73 deletion in non-hematopoietic cells results in higher Th1 cell responses and marked joint damage in a mouse model of collagen induced arthritis [52].

Recent investigations have also reported a correlation between CD39/ENTPD1 and CD73 expression on CD4^+^ T-cell-derived microparticles (MPs) and disease activity. High levels of CD4^+^CD161^+^CD39^+^ MPs positively correlate, while CD4^+^CD39^+^CD73^+^ MPs have a negative correlation with RA activity. It is feasible that MPs with differential phenotypes might serve as biomarkers for disease monitoring [53].

In PBMCs from RA patients, the disease-related high concentration of pro-inflammatory cytokines induces expression of NF-κB and CREB as well as activation of the PI3K/Akt pathway, leading to A2R and A3R upregulation [54,55,56]. Adenosine receptors upregulation inversely correlates with the disease activity score and is associated with decreases in TNF-α, IL-1β and IL-6 levels [56]. Further, agonist-induced A2AR and A3R activation in RA-derived lymphocytes results in inhibition of NF-κB signaling and reduction in metalloproteinases [57].

### 2.3. Type 1 Diabetes

Type 1 diabetes is considered an autoimmune disorder targeting the insulin-producing pancreatic β-cells. Genetic predisposition along with environmental factors have been proposed as disease triggers. No cure is currently available, and the goal of current treatments is to control blood sugar levels by insulin replacement therapies with the view of the prevention of vascular, neurological and other complications.

Experimental evidence provided by murine models suggests that high levels of CD39, in association with high A2AR and A2BR expression in T-helper cells confer protection from streptozotocin-induced diabetes [58]. Further studies have revealed that suppression of pro-inflammatory cytokine release is predominantly mediated upon A2BR engagement [59]. Positive correlations between low CD39/ENTPD1 levels and disease activity has been observed in type 1 diabetic children, suggesting a potential compromise in Treg function [60].

Toll-like-receptor-9 (TLR9) deficiency has been associated with CD73-mediated beneficial effects. TLR9^−/−^ non-obese diabetic mice display higher levels of CD73 on CD4^+^ T-cells, lower levels of pro-inflammatory cytokines and increased anti-inflammatory cytokine production; these all being linked to protection against diabetes [61].

### 2.4. Autoimmune Hepatitis and Cholestatic Liver Disorders

There is growing evidence that defects in ectonucleotidase activity and impaired P1 receptor levels contribute to loss of immunotolerance in autoimmune liver disorders (Figure 1B). In type-1 autoimmune hepatitis (AIH-1), CD39^+^ Treg cells are decreased in number and display impaired ability to suppress IL-17 production by CD4^+^ effectors. Reduced CD39^+^ Treg frequencies might result from cell instability upon pro-inflammatory challenge, with consequent increased rate of conversion into effector lymphocytes [62]. In both AIH and autoimmune sclerosing cholangitis, there is a decrease in CD39^+^ Th17-cell numbers, associated with impaired overall cell-associated ADPase activity and lower A2AR expression [63]. Further, in acutely presenting, untreated AIH patients there is low ratio between Tregs and a specific NK subset with activated effector phenotype [64]. In this same study, Tregs were found to display an activated memory phenotype and exhibit signs of exhaustion, including increased CTLA-4 and PD-1 receptor levels, as well as decreased ability to limit pro-inflammatory responses [64].

Murine studies conducted in the context of experimental cholestasis have proposed a pathogenic role for A1R signaling in mediating liver injury, as lack of A1R limits the efflux of toxic biliary constituents through the biliary excretory route [65]. On the other hand, A2BR activation in mouse cholangiocytes, promotes IL-6 expression via cAMP and Ca^2+^ signaling, favoring cholangiocyte survival during biliary cirrhosis [66]. Patients affected by primary biliary cholangitis exhibit dramatic phenotypic alterations in CD8^+^ Tregs, as reflected by increased levels of CD127 and lower CD39/ENTPD1 expression that correlate with lower responsiveness to IL-10 [67].

There is evidence that mice deficient in the ABCB4/multi-drug-resistant-protein2 (MDR2) transporter protein—an experimental model for human primary sclerosing cholangitis (PSC)—show expanded intrahepatic CD8^+^ lymphocytes that positively correlate with biliary injury and fibrosis [68]. Depletion of CD8^+^ cells in MDR2^−/−^/CD39^−/−^ mice attenuates hepatobiliary injury and fibrosis; while administration of αβ-ATP into Mdr2^−/−^/CD39 WT mice mirrors the phenotype of MDR2^−/−^/CD39^−/−^ mice [69].

### 2.5. Inflammatory Bowel Disease

A number of studies have demonstrated close links between ectonucleotidases or P1 receptor signaling and the immunopathogenesis of inflammatory bowel disease (IBD); and, specifically, in Crohn’s disease and ulcerative colitis. Th17-cells are pivotal players in IBD pathogenesis and require eATP for complete differentiation [70]. High levels of CD39/ENTPD1 expression endow Th17-cells with suppressor phenotypes and immunoregulatory function. Crohn’s patients present lower frequencies of these SupTh17 lymphocytes, when compared to healthy subjects [71]. In mice, CD39/ENTPD1 deletion exacerbates dextran-sulfate-sodium (DSS)-induced experimental colitis [72]. Furthermore, the presence of genetic polymorphisms of *ENTPD1* and also levels of expression of CD39 on Tregs, have been associated with increased susceptibility to Crohn’s disease in humans and in predicting the response to immunomodulatory therapy, respectively [72,73].

Importantly, in IBD patients during clinical and endoscopic remission, peripheral blood-derived Tregs express higher CD39 levels [73]; further, therapeutic drug levels in responders are associated with higher CD39 expression in FOXP3^+^ Tregs [73].

Regulatory effects of unconjugated bilirubin (UCB) are detectable in healthy human and WT murine Th17-cells, significantly ameliorating DSS-colitis in vivo [7]. SupTh17-cells can be induced upon exposure of conventional Th17-cells to certain metabolites e.g., UCB that boost CD39/ENTPD1 expression via AhR engagement [7,8]. In Crohn’s disease, however, Th17-cells display lower AhR expression and higher levels of hypoxia-inducible-factor-1alpha (HIF-1α), known to inhibit AhR levels [23] and signaling [8]. Increases in HIF-1α results in heightened expression of ATP-binding-cassette transporters that also induce extracellular efflux of immunometabolites like UCB [8], therefore dampening immunosuppressive potential.

AhR activation, by the relatively non-toxic agonist 2-(1′H-indole-3′-carbonyl)-thiazole-4-carboxylic acid methyl ester (ITE), increases CD39, IL-10 as well as granzyme B expression in Tregs [9]. High CD39 levels in Foxp3^+^ Tregs have been associated with Crohn’s remission, also in response to anti-TNF-α treatment [73]. Conversely, co-expression of CD39/ENTPD1 and CD161 in Th17-cells correlates with a pro-inflammatory cellular phenotype that is upregulated in Crohn’s patients [74]. Exposure to CD3/CD28 stimulation induces CD39/ENTPD1 also in CD8^+^ T-cells, an effector subset involved in IBD pathogenesis. CD39^+^CD8^+^ T-cells thwart IFNγ production by CD39^−^CD8^+^ T-cells, this effect being mediated by A2AR in a paracrine manner [75].

Other studies have indicated a protective role for CD73, the lack of which leads to heightened susceptibility to DSS colitis, marked weight loss, gut permeability and accumulation of pro-inflammatory cytokines [76] in mice. However, CD73 expression on effector CD4^+^ cells has been also associated with a pro-inflammatory phenotype. Pro-inflammatory CD73^+^CD4^+^ T-cells with a Th17 signature are enriched in peripheral blood and lamina propria of IBD patients [77], suggesting compensatory mechanisms that are activated during active inflammation.

Studies on P1 signaling have demonstrated that A2AR activation reduces intestinal inflammation, TNF, IFNγ and IL-4 levels as well as colonic inflammatory cell infiltration in vivo [78]. Conversely, global A2BR deletion protects from inflammatory damage induced by DSS, 2,4,6-trinitrobenzene sulfonic acid (TNBS), *Salmonella typhimurium* and IL-8-induced colitis [79]. However, recent investigations on mice with A2BR conditional deletion on vascular endothelial or intestinal epithelial cells attributed the ability to reduce colonic inflammation only to A2BR expressed on epithelial cells. Adenosine can also promote intestinal epithelial barrier restoration, especially during disease remission. The effect is mediated by the nucleoside transporters 1 and 2 that remove adenosine from the extracellular space upon A2BR activation [80].

### 2.6. Multiple Sclerosis

Ectonucleotidase activity and adenosine signaling exhibit protective properties also in multiple sclerosis (MS), a neuroinflammatory autoimmune disorder driven by pathogenic T-cells specific for myelin antigens in the central nervous system (CNS) [81,82].

In mice with experimental autoimmune encephalomyelitis (EAE), the murine model for MS, administration of capsular polysaccharide A (PSA), the symbiosis factor for human intestinal commensal *Bacteroides fragilis*, elicits immunotolerance by promoting expansion and accumulation of CD39^+^CD4^+^ cells in CNS lymphoid-draining sites [83]. PSA-mediated CD39^+^CD4^+^ T-cell expansion is driven by TLR2 signaling and the protective effect is completely abrogated in the absence of ENTPD1/CD39 [84].

ENTPD1/CD39^+^ expression in human regulatory cells has been closely associated to MS different stages. CD25^+^Foxp3^+^CD39^+^ Treg cells are impaired in peripheral blood of MS patients and cell frequency is further reduced in the remitting/relapsing form of the disease [82,85]. Around 40% of relapsing-remitting MS cases present with apparent Th17-cell expansion with a positive correlation between Th17-cell numbers and ENTPD1/CD39^+^ Treg frequencies during remission but not during relapse. These studies suggest that dysregulation of the Th17/CD39^+^ Treg functional balance may contribute to exacerbation of the disease [86].

A1R regulates IL-6 and TNF-α expression. Interestingly, reduced A1R levels are detectable in the microglia and peripheral blood of MS patients [87,88,89]; while A1R null mice develop a more severe form of EAE, characterized by increased pro-inflammatory gene expression, microglial activation and demyelination as compared to WT controls [87]. Accordingly, caffeine administration increases A1R expression and improves animal clinical condition further supporting the receptor protective effect [87].

## 3. Therapeutic Implications

**Figure 2:**
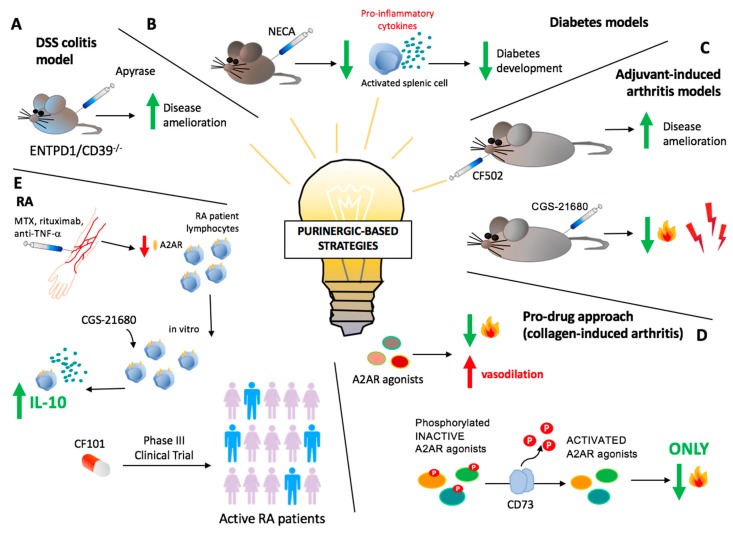
Purinergic-based therapeutic strategies. (**A**) Administration of exogenous apyrase strongly ameliorates dextran-sulfate-sodium (DSS) colitis in ENTPD1/CD39^−/−^ mice. (**B**) In experimental murine diabetes models, treatment with the nonselective adenosine receptor agonist 5′-*N*-ethylcarboxamidoadenosine (NECA) prevents diabetes development by suppressing expression of pro-inflammatory cytokines by activated splenic cells. (**C**) In rat models of adjuvant-induced arthritis, administration of the A2AR agonist CGS 21680 shows anti-inflammatory and analgesic properties. Similarly, orally administrated, low doses of the A3R agonist CF502 significantly ameliorate clinical condition. (**D**) On the other hand, A2AR agonists have also been described as highly effective vasodilators, having, as a major side effect, hypotension. An improved and promising therapeutic approach might be the use of phosphorylated A2AR agonists (prodrugs) that need the ecto-5′-nucleotidase(CD73)-mediated de-phosphorylation in order to be activated. As an example, in a murine model of collagen-induced arthritis, the prodrug 2-(cyclohexylethylthio)adenosine 5′-monophosphate (chet-AMP), showed potent immunosuppressive properties, with negligible vasodilatory side effects. (**E**) In rheumatoid arthritis (RA) patients, anti-TNF-α agents, rituximab or methotrexate (MTX) reduce the compensatory and protective increase in A2AR expression in peripheral blood lymphocytes. However, studies reveal that in vitro stimulation with the receptor agonist CGS 21680 significantly promotes immunoregulatory responses, increasing IL-10 production. Administration of CF101, an A3R specific agonist, is currently being tested in clinical trials.

Modulation of adenosine signaling represents a promising therapeutic tool for several autoimmune diseases (Figure 2). In previous studies, administration of apyrase, which has ectoenzymatic activity comparable to CD39, strongly ameliorated DSS colitis in ENTPD1/CD39^−/−^ mice [72]. Protective effects of apyrase were also confirmed in subsequent studies conducted in the context of already established DSS colitis [90].

Therapeutic strategies modulating the purinergic signaling involve direct targeting of adenosine receptors, either by administration of receptors agonists, like adenosine, or by pharmacological antagonization (Figure 2). Although most of these approaches are still under evaluation, some have been already applied to the clinical setting, like the pharmacological preconditioning of explanted livers with adenosine solution, which prevents ischemic damage consequent to organ reperfusion [91,92,93]. In two different experimental murine models, administration of the nonselective adenosine receptor agonist 5′-*N*-ethylcarboxamidoadenosine (NECA), significantly prevented diabetes development by suppressing expression of pro-inflammatory cytokines by activated splenic cells, including Th1 cells [59]. Experiments conducted in primary murine myeloid cells showed that A2AR activation regulates bone turnover inhibiting osteoclast differentiation. The effect is mediated by PKA-dependent inhibition of NF-κB nuclear translocation. This supports the use of A2AR agonists for targeting inflammatory conditions affecting the bones, including RA [94]. Administration of the A2AR agonist CGS 21680 results in anti-inflammatory as well as analgesic properties in a rat model of adjuvant-induced arthritis. A2AR expression is upregulated in circulating lymphocytes of RA and MS patients, probably as a compensatory response to counteract inflammation [95,96,97]. The increased A2AR expression in RA lymphocytes is gradually reduced by anti-TNF-α agents like rituximab or MTX; however, in vitro stimulation with the receptor agonist CGS 21680 significantly increases IL-10 production [97]. Likewise, A2AR activation inhibits cell proliferation and pro-inflammatory cytokine production in lymphocytes from MS patients [95].

A2AR plays a pivotal role also in modulating the inflammatory response in hypoxic conditions, especially in the acute setting. It is well established that lack of oxygen induces adenosine release in several body compartments [98] and there is now evidence that, in murine models of T-cell-mediated acute hepatitis, A2AR mediates the hypoxia-induced protection from liver damage. A2AR deletion and pharmacological antagonization significantly abrogate the hypoxia-mediated anti-inflammatory effects in acute liver tissue injury [99].

Additional studies have reported that synthesized phosphorylated A2AR agonists (prodrugs) that need the ecto-5′-nucleotidase (CD73)-mediated de-phosphorylation in order to be activated, were tested in a murine model of collagen-induced arthritis. The prodrug effect was evaluated also upon inhibition of CD73 and A2AR. Among the tested compounds, 2-(cyclohexylethylthio)adenosine 5′-monophosphate (chet-AMP) showed potent immunosuppressive properties, with negligible vasodilatory side effects, supporting the use of phosphorylated A2AR agonists for the specific treatment of inflammation [100]. Encouraging results were also obtained with a non-absorbable, locally active A2AR agonist, named as 4-(2-ethyl)-benzenesulfonic acid (7, PSB-0777) that was recently proposed as novel treatment for inflammatory bowel syndrome. Ex vivo treatment of rat ileum/jejunum preparations with PSB-0777 alone or in combination with A2BR antagonists, significantly ameliorated the impaired acetylcholine-induced contractions induced by TNBS [101].

Further, in vitro treatment of fibroblast-like synoviocytes with CF502, a selective A3R agonist with high affinity for the human subtype, markedly inhibited cell proliferation. Moreover, in a rat experimental model of adjuvant-induced arthritis, oral administration of low doses of CF502 significantly ameliorated the clinical phenotype [102]. Previous studies have shown that in a phase II clinical trial in patients with active RA, administration of CF101—a specific A3R agonist—resulted in amelioration of disease that, however, did not reach statistical significance [103,104]. In the same study, levels of A3R on patients’ PMBCs at baseline correlated directly with clinical response to CF101, suggesting A3R as a predictive therapeutic biomarker [103,104]. In subsequent investigations, CF101 was reported to reduce pannus formation and lymphocyte infiltration in rats with osteoarthritis by deregulating NF-κB [105]. CF101 is currently being tested in a phase III clinical trial in patients with active RA (NCT02647762).

In experimental colitis, treatment with the A3R agonist *N*^6^-(3-iodobenzyl)-adenosine-5-*N*-methyluronamide (IB-MECA) significantly prevented colitis-induced gene dysregulation, weight loss and gut injury [106].

## 4. Concluding Remarks

The Janus-like nature of the purinergic signaling involves release of eATP that boosts inflammation; and also results in eATP hydrolysis, which leads to the generation of adenosine that suppresses the immune response. Dysregulation of the ATP/adenosine balance occurs in autoimmune conditions and positively correlates with disease severity and progression. Due to the high levels of immunological heterogeneity of these conditions, current treatments directly targeting the immune response are often poorly effective and are associated with significant side effects.

Modulation of the purinergic response could therefore be a novel approach to improve current therapeutics. Promising molecular candidates have been already identified in ectonucleotidases (especially ENTPD1/CD39 and CD73) and P1 receptor agonists. Pre-clinical data support the pharmacological induction of ENTPD1/CD39 expression as well as the stimulation of A2AR and A3R as potential ways of treatment. We propose that purinergic-based strategies, alone or in combination with current treatments, might represent strong adjunctive therapeutics to help dampen inflammation and interfere with disease progression without untoward toxicity.

## References

[1] Burnstock G. (2014). Purinergic signalling: From discovery to current developments. Exp. Physiol..

[2] Di Virgilio F., Vuerich M. (2015). Purinergic signaling in the immune system. Auton. Neurosci..

[3] Eltzschig H.K., Sitkovsky M.V., Robson S.C. (2012). Purinergic signaling during inflammation. N. Engl. J. Med..

[4] Longhi M.S., Moss A., Jiang Z.G., Robson S.C. (2017). Purinergic signaling during intestinal inflammation. J. Mol. Med..

[5] Yegutkin G.G. (2014). Enzymes involved in metabolism of extracellular nucleotides and nucleosides: Functional implications and measurement of activities. Crit. Rev. Biochem. Mol. Biol..

[6] Allard B., Longhi M.S., Robson S.C., Stagg J. (2017). The ectonucleotidases CD39 and CD73: Novel checkpoint inhibitor targets. Immunol. Rev..

[7] Longhi M.S., Vuerich M., Kalbasi A., Kenison J.E., Yeste A., Csizmadia E., Vaughn B., Feldbrugge L., Mitsuhashi S., Wegiel B. (2017). Bilirubin suppresses Th17 immunity in colitis by upregulating CD39. JCI Insight.

[8] Xie A., Robles R.J., Mukherjee S., Zhang H., Feldbrugge L., Csizmadia E., Wu Y., Enjyoji K., Moss A.C., Otterbein L.E. (2018). HIF-1alpha-induced xenobiotic transporters promote Th17 responses in Crohn’s disease. J. Autoimmun..

[9] Goettel J.A., Gandhi R., Kenison J.E., Yeste A., Murugaiyan G., Sambanthamoorthy S., Griffith A.E., Patel B., Shouval D.S., Weiner H.L. (2016). AHR Activation Is Protective against Colitis Driven by T Cells in Humanized Mice. Cell Rep..

[10] Deaglio S., Dwyer K.M., Gao W., Friedman D., Usheva A., Erat A., Chen J.F., Enjyoji K., Linden J., Oukka M. (2007). Adenosine generation catalyzed by CD39 and CD73 expressed on regulatory T cells mediates immune suppression. J. Exp. Med..

[11] Dwyer K.M., Hanidziar D., Putheti P., Hill P.A., Pommey S., McRae J.L., Winterhalter A., Doherty G., Deaglio S., Koulmanda M. (2010). Expression of CD39 by human peripheral blood CD4^+^ CD25^+^ T cells denotes a regulatory memory phenotype. Am. J. Transplant..

[12] Alam M.S., Kurtz C.C., Rowlett R.M., Reuter B.K., Wiznerowicz E., Das S., Linden J., Crowe S.E., Ernst P.B. (2009). CD73 is expressed by human regulatory T helper cells and suppresses proinflammatory cytokine production and Helicobacter felis-induced gastritis in mice. J. Infect. Dis..

[13] Kobie J.J., Shah P.R., Yang L., Rebhahn J.A., Fowell D.J., Mosmann T.R. (2006). T regulatory and primed uncommitted CD4 T cells express CD73, which suppresses effector CD4 T cells by converting 5’-adenosine monophosphate to adenosine. J. Immunol..

[14] Bao R., Hou J., Li Y., Bian J., Deng X., Zhu X., Yang T. (2016). Adenosine promotes Foxp3 expression in Treg cells in sepsis model by activating JNK/AP-1 pathway. Am. J. Transl. Res..

[15] Schenk U., Frascoli M., Proietti M., Geffers R., Traggiai E., Buer J., Ricordi C., Westendorf A.M., Grassi F. (2011). ATP inhibits the generation and function of regulatory T cells through the activation of purinergic P2X receptors. Sci. Signal..

[16] Huang S., Apasov S., Koshiba M., Sitkovsky M. (1997). Role of A2a extracellular adenosine receptor-mediated signaling in adenosine-mediated inhibition of T-cell activation and expansion. Blood.

[17] Liao H., Hyman M.C., Baek A.E., Fukase K., Pinsky D.J. (2010). cAMP/CREB-mediated transcriptional regulation of ectonucleoside triphosphate diphosphohydrolase 1 (CD39) expression. J. Biol. Chem..

[18] Kinsey G.R., Huang L., Jaworska K., Khutsishvili K., Becker D.A., Ye H., Lobo P.I., Okusa M.D. (2012). Autocrine adenosine signaling promotes regulatory T cell-mediated renal protection. J. Am. Soc. Nephrol..

[19] Ohta A., Kini R., Ohta A., Subramanian M., Madasu M., Sitkovsky M. (2012). The development and immunosuppressive functions of CD4^+^ CD25^+^ FoxP3^+^ regulatory T cells are under influence of the adenosine-A2A adenosine receptor pathway. Front. Immunol..

[20] Liang D., Woo J.I., Shao H., Born W.K., O’Brien R.L., Kaplan H.J., Sun D. (2018). Ability of gammadelta T cells to modulate the Foxp3 T cell response is dependent on adenosine. PLoS ONE.

[21] Ring S., Pushkarevskaya A., Schild H., Probst H.C., Jendrossek V., Wirsdorfer F., Ledent C., Robson S.C., Enk A.H., Mahnke K. (2015). Regulatory T cell-derived adenosine induces dendritic cell migration through the Epac-Rap1 pathway. J. Immunol..

[22] Ehrentraut H., Westrich J.A., Eltzschig H.K., Clambey E.T. (2012). Adora2b adenosine receptor engagement enhances regulatory T cell abundance during endotoxin-induced pulmonary inflammation. PLoS ONE.

[23] Mascanfroni I.D., Takenaka M.C., Yeste A., Patel B., Wu Y., Kenison J.E., Siddiqui S., Basso A.S., Otterbein L.E., Pardoll D.M. (2015). Metabolic control of type 1 regulatory T cell differentiation by AHR and HIF1-alpha. Nat. Med..

[24] McGeachy M.J., Bak-Jensen K.S., Chen Y., Tato C.M., Blumenschein W., McClanahan T., Cua D.J. (2007). TGF-beta and IL-6 drive the production of IL-17 and IL-10 by T cells and restrain T(H)-17 cell-mediated pathology. Nat. Immunol..

[25] Flores-Santibanez F., Fernandez D., Meza D., Tejon G., Vargas L., Varela-Nallar L., Arredondo S., Guixe V., Rosemblatt M., Bono M.R. (2015). CD73-mediated adenosine production promotes stem cell-like properties in mouse Tc17 cells. Immunology.

[26] Bono M.R., Fernandez D., Flores-Santibanez F., Rosemblatt M., Sauma D. (2015). CD73 and CD39 ectonucleotidases in T cell differentiation: Beyond immunosuppression. FEBS Lett..

[27] Gupta P.K., Godec J., Wolski D., Adland E., Yates K., Pauken K.E., Cosgrove C., Ledderose C., Junger W.G., Robson S.C. (2015). CD39 Expression Identifies Terminally Exhausted CD8^+^ T Cells. PLoS Pathog..

[28] Hindupur S.K., Gonzalez A., Hall M.N. (2015). The opposing actions of target of rapamycin and AMP-activated protein kinase in cell growth control. Cold Spring Harb. Perspect. Biol..

[29] Farez M.F., Mascanfroni I.D., Mendez-Huergo S.P., Yeste A., Murugaiyan G., Garo L.P., Balbuena Aguirre M.E., Patel B., Ysrraelit M.C., Zhu C. (2015). Melatonin Contributes to the Seasonality of Multiple Sclerosis Relapses. Cell.

[30] Mann E.H., Chambers E.S., Chen Y.H., Richards D.F., Hawrylowicz C.M. (2015). 1alpha,25-dihydroxyvitamin D3 acts via transforming growth factor-beta to up-regulate expression of immunosuppressive CD73 on human CD4^+^ Foxp3^−^ T cells. Immunology.

[31] Regateiro F.S., Howie D., Nolan K.F., Agorogiannis E.I., Greaves D.R., Cobbold S.P., Waldmann H. (2011). Generation of anti-inflammatory adenosine by leukocytes is regulated by TGF-beta. Eur. J. Immunol..

[32] Antonioli L., Blandizzi C., Pacher P., Hasko G. (2013). Immunity, inflammation and cancer: A leading role for adenosine. Nat. Rev. Cancer.

[33] Gsandtner I., Charalambous C., Stefan E., Ogris E., Freissmuth M., Zezula J. (2005). Heterotrimeric G protein-independent signaling of a G protein-coupled receptor. Direct binding of ARNO/cytohesin-2 to the carboxyl terminus of the A2A adenosine receptor is necessary for sustained activation of the ERK/MAP kinase pathway. J. Biol. Chem..

[34] Peter D., Jin S.L., Conti M., Hatzelmann A., Zitt C. (2007). Differential expression and function of phosphodiesterase 4 (PDE4) subtypes in human primary CD4^+^ T cells: Predominant role of PDE4D. J. Immunol..

[35] Keuerleber S., Gsandtner I., Freissmuth M. (2011). From cradle to twilight: The carboxyl terminus directs the fate of the A(2A)-adenosine receptor. Biochim. Biophys. Acta.

[36] Kreth S., Ledderose C., Kaufmann I., Groeger G., Thiel M. (2008). Differential expression of 5′-UTR splice variants of the adenosine A2A receptor gene in human granulocytes: Identification, characterization, and functional impact on activation. FASEB J..

[37] Zhang L., Yang N., Wang S., Huang B., Li F., Tan H., Liang Y., Chen M., Li Y., Yu X. (2011). Adenosine 2A receptor is protective against renal injury in MRL/lpr mice. Lupus.

[38] Knight J.S., Mazza L.F., Yalavarthi S., Sule G., Ali R.A., Hodgin J.B., Kanthi Y., Pinsky D.J. (2018). Ectonucleotidase-Mediated Suppression of Lupus Autoimmunity and Vascular Dysfunction. Front. Immunol..

[39] Loza M.J., Anderson A.S., O’Rourke K.S., Wood J., Khan I.U. (2011). T-cell specific defect in expression of the NTPDase CD39 as a biomarker for lupus. Cell Immunol..

[40] Kammer G.M., Birch R.E., Polmar S.H. (1983). Impaired immunoregulation in systemic lupus erythematosus: Defective adenosine-induced suppressor T lymphocyte generation. J. Immunol..

[41] Schultz L.A., Kammer G.M., Rudolph S.A. (1988). Characterization of the human T lymphocyte adenosine receptor: Comparison of normal and systemic lupus erythematosus cells. FASEB J..

[42] Bortoluzzi A., Vincenzi F., Govoni M., Padovan M., Ravani A., Borea P.A., Varani K. (2016). A2A adenosine receptor upregulation correlates with disease activity in patients with systemic lupus erythematosus. Arthritis Res. Ther..

[43] McInnes I.B., Schett G. (2011). The pathogenesis of rheumatoid arthritis. N. Engl. J. Med..

[44] Peres R.S., Donate P.B., Talbot J., Cecilio N.T., Lobo P.R., Machado C.C., Lima K.W.A., Oliveira R.D., Carregaro V., Nakaya H.I. (2018). TGF-beta signalling defect is linked to low CD39 expression on regulatory T cells and methotrexate resistance in rheumatoid arthritis. J. Autoimmun..

[45] Peres R.S., Liew F.Y., Talbot J., Carregaro V., Oliveira R.D., Almeida S.L., Franca R.F., Donate P.B., Pinto L.G., Ferreira F.I. (2015). Low expression of CD39 on regulatory T cells as a biomarker for resistance to methotrexate therapy in rheumatoid arthritis. Proc. Natl. Acad. Sci. USA.

[46] Hider S.L., Thomson W., Mack L.F., Armstrong D.J., Shadforth M., Bruce I.N. (2008). Polymorphisms within the adenosine receptor 2a gene are associated with adverse events in RA patients treated with MTX. Rheumatology.

[47] Veras F.P., Peres R.S., Saraiva A.L., Pinto L.G., Louzada-Junior P., Cunha T.M., Paschoal J.A., Cunha F.Q., Alves-Filho J.C. (2015). Fructose 1,6-bisphosphate, a high-energy intermediate of glycolysis, attenuates experimental arthritis by activating anti-inflammatory adenosinergic pathway. Sci. Rep..

[48] Thiolat A., Semerano L., Pers Y.M., Biton J., Lemeiter D., Portales P., Quentin J., Jorgensen C., Decker P., Boissier M.C. (2014). Interleukin-6 receptor blockade enhances CD39^+^ regulatory T cell development in rheumatoid arthritis and in experimental arthritis. Arthritis Rheumatol..

[49] Nie H., Zheng Y., Li R., Guo T.B., He D., Fang L., Liu X., Xiao L., Chen X., Wan B. (2013). Phosphorylation of FOXP3 controls regulatory T cell function and is inhibited by TNF-alpha in rheumatoid arthritis. Nat. Med..

[50] Dos Santos Jaques J.A., Becker L.V., Souza Vdo C., Leal C.A., Bertoldo T.M., de Vargas Pinheiro K., Morsch V.M., Schetinger M.R., Leal D.B. (2013). Activities of enzymes that hydrolyze adenine nucleotides in lymphocytes from patients with rheumatoid arthritis. Cell Biochem. Funct..

[51] Herrath J., Chemin K., Albrecht I., Catrina A.I., Malmstrom V. (2014). Surface expression of CD39 identifies an enriched Treg-cell subset in the rheumatic joint, which does not suppress IL-17A secretion. Eur. J. Immunol..

[52] Chrobak P., Charlebois R., Rejtar P., El Bikai R., Allard B., Stagg J. (2015). CD73 plays a protective role in collagen-induced arthritis. J. Immunol..

[53] Fan W., Wang W., Wu J., Ma L., Guo J. (2017). Identification of CD4^+^ T-cell-derived CD161^+^ CD39^+^ and CD39^+^CD73^+^ microparticles as new biomarkers for rheumatoid arthritis. Biomark. Med..

[54] Madi L., Cohen S., Ochayin A., Bar-Yehuda S., Barer F., Fishman P. (2007). Overexpression of A3 adenosine receptor in peripheral blood mononuclear cells in rheumatoid arthritis: Involvement of nuclear factor-kappaB in mediating receptor level. J. Rheumatol..

[55] Ochaion A., Bar-Yehuda S., Cohen S., Barer F., Patoka R., Amital H., Reitblat T., Reitblat A., Ophir J., Konfino I. (2009). The anti-inflammatory target A(3) adenosine receptor is over-expressed in rheumatoid arthritis, psoriasis and Crohn’s disease. Cell Immunol..

[56] Varani K., Padovan M., Vincenzi F., Targa M., Trotta F., Govoni M., Borea P.A. (2011). A2A and A3 adenosine receptor expression in rheumatoid arthritis: Upregulation, inverse correlation with disease activity score and suppression of inflammatory cytokine and metalloproteinase release. Arthritis Res. Ther..

[57] Ravani A., Vincenzi F., Bortoluzzi A., Padovan M., Pasquini S., Gessi S., Merighi S., Borea P.A., Govoni M., Varani K. (2017). Role and Function of A2A and A(3) Adenosine Receptors in Patients with Ankylosing Spondylitis, Psoriatic Arthritis and Rheumatoid Arthritis. Int. J. Mol. Sci..

[58] Chia J.S., McRae J.L., Thomas H.E., Fynch S., Elkerbout L., Hill P., Murray-Segal L., Robson S.C., Chen J.F., d’Apice A.J. (2013). The protective effects of CD39 overexpression in multiple low-dose streptozotocin-induced diabetes in mice. Diabetes.

[59] Nemeth Z.H., Bleich D., Csoka B., Pacher P., Mabley J.G., Himer L., Vizi E.S., Deitch E.A., Szabo C., Cronstein B.N. (2007). Adenosine receptor activation ameliorates type 1 diabetes. FASEB J..

[60] Akesson K., Tompa A., Ryden A., Faresjo M. (2015). Low expression of CD39^+^/CD45RA^+^ on regulatory T cells (T_reg_) cells in type 1 diabetic children in contrast to high expression of CD101^+^/CD129^+^ on T_reg_ cells in children with coeliac disease. Clin. Exp. Immunol..

[61] Tai N., Wong F.S., Wen L. (2013). TLR9 deficiency promotes CD73 expression in T cells and diabetes protection in nonobese diabetic mice. J. Immunol..

[62] Grant C.R., Liberal R., Holder B.S., Cardone J., Ma Y., Robson S.C., Mieli-Vergani G., Vergani D., Longhi M.S. (2014). Dysfunctional CD39(POS) regulatory T cells and aberrant control of T-helper type 17 cells in autoimmune hepatitis. Hepatology.

[63] Liberal R., Grant C.R., Ma Y., Csizmadia E., Jiang Z.G., Heneghan M.A., Yee E.U., Mieli-Vergani G., Vergani D., Robson S.C. (2016). CD39 mediated regulation of Th17-cell effector function is impaired in juvenile autoimmune liver disease. J. Autoimmun..

[64] Jeffery H.C., Braitch M.K., Bagnall C., Hodson J., Jeffery L.E., Wawman R.E., Wong L.L., Birtwistle J., Bartlett H., Lohse A.W. (2018). Changes in natural killer cells and exhausted memory regulatory T Cells with corticosteroid therapy in acute autoimmune hepatitis. Hepatol. Commun..

[65] Yang P., Chen P., Wang T., Zhan Y., Zhou M., Xia L., Cheng R., Guo Y., Zhu L., Zhang J. (2013). Loss of A(1) adenosine receptor attenuates alpha-naphthylisothiocyanate-induced cholestatic liver injury in mice. Toxicol. Sci..

[66] Lavoie E.G., Fausther M., Goree J.R., Dranoff J.A. (2017). The Cholangiocyte Adenosine-IL-6 Axis Regulates Survival During Biliary Cirrhosis. Gene Expr..

[67] Bernuzzi F., Fenoglio D., Battaglia F., Fravega M., Gershwin M.E., Indiveri F., Ansari A.A., Podda M., Invernizzi P., Filaci G. (2010). Phenotypical and functional alterations of CD8 regulatory T cells in primary biliary cirrhosis. J. Autoimmun..

[68] Taylor A.E., Carey A.N., Kudira R., Lages C.S., Shi T., Lam S., Karns R., Simmons J., Shanmukhappa K., Almanan M. (2018). Interleukin 2 Promotes Hepatic Regulatory T Cell Responses and Protects From Biliary Fibrosis in Murine Sclerosing Cholangitis. Hepatology.

[69] Peng Z.W., Rothweiler S., Wei G., Ikenaga N., Liu S.B., Sverdlov D.Y., Vaid K.A., Longhi M.S., Kuang M., Robson S.C. (2017). The ectonucleotidase ENTPD1/CD39 limits biliary injury and fibrosis in mouse models of sclerosing cholangitis. Hepatol. Commun..

[70] Fujino S., Andoh A., Bamba S., Ogawa A., Hata K., Araki Y., Bamba T., Fujiyama Y. (2003). Increased expression of interleukin 17 in inflammatory bowel disease. Gut.

[71] Longhi M.S., Moss A., Bai A., Wu Y., Huang H., Cheifetz A., Quintana F.J., Robson S.C. (2014). Characterization of human CD39^+^ Th17 cells with suppressor activity and modulation in inflammatory bowel disease. PLoS ONE.

[72] Friedman D.J., Kunzli B.M., YI A.R., Sevigny J., Berberat P.O., Enjyoji K., Csizmadia E., Friess H., Robson S.C. (2009). From the Cover: CD39 deletion exacerbates experimental murine colitis and human polymorphisms increase susceptibility to inflammatory bowel disease. Proc. Natl. Acad. Sci. USA.

[73] Gibson D.J., Elliott L., McDermott E., Tosetto M., Keegan D., Byrne K., Martin S.T., Rispens T., Cullen G., Mulcahy H.E. (2015). Heightened Expression of CD39 by Regulatory T Lymphocytes Is Associated with Therapeutic Remission in Inflammatory Bowel Disease. Inflamm. Bowel Dis..

[74] Bai A., Moss A., Kokkotou E., Usheva A., Sun X., Cheifetz A., Zheng Y., Longhi M.S., Gao W., Wu Y. (2014). CD39 and CD161 modulate Th17 responses in Crohn’s disease. J. Immunol..

[75] Bai A., Moss A., Rothweiler S., Longhi M.S., Wu Y., Junger W.G., Robson S.C. (2015). NADH oxidase-dependent CD39 expression by CD8^+^ T cells modulates interferon gamma responses via generation of adenosine. Nat. Commun..

[76] Bynoe M.S., Waickman A.T., Mahamed D.A., Mueller C., Mills J.H., Czopik A. (2012). CD73 is critical for the resolution of murine colonic inflammation. J. Biomed. Biotechnol..

[77] Doherty G.A., Bai A., Hanidziar D., Longhi M.S., Lawlor G.O., Putheti P., Csizmadia E., Nowak M., Cheifetz A.S., Moss A.C. (2012). CD73 is a phenotypic marker of effector memory Th17 cells in inflammatory bowel disease. Eur. J. Immunol..

[78] Odashima M., Bamias G., Rivera-Nieves J., Linden J., Nast C.C., Moskaluk C.A., Marini M., Sugawara K., Kozaiwa K., Otaka M. (2005). Activation of A2A adenosine receptor attenuates intestinal inflammation in animal models of inflammatory bowel disease. Gastroenterology.

[79] Kolachala V.L., Vijay-Kumar M., Dalmasso G., Yang D., Linden J., Wang L., Gewirtz A., Ravid K., Merlin D., Sitaraman S.V. (2008). A2B adenosine receptor gene deletion attenuates murine colitis. Gastroenterology.

[80] Aherne C.M., Collins C.B., Rapp C.R., Olli K.E., Perrenoud L., Jedlicka P., Bowser J.L., Mills T.W., Karmouty-Quintana H., Blackburn M.R. (2018). Coordination of ENT2-dependent adenosine transport and signaling dampens mucosal inflammation. JCI Insight.

[81] Martin R., Jaraquemada D., Flerlage M., Richert J., Whitaker J., Long E.O., McFarlin D.E., McFarland H.F. (1990). Fine specificity and HLA restriction of myelin basic protein-specific cytotoxic T cell lines from multiple sclerosis patients and healthy individuals. J. Immunol..

[82] Fletcher J.M., Lonergan R., Costelloe L., Kinsella K., Moran B., O’Farrelly C., Tubridy N., Mills K.H. (2009). CD39^+^Foxp3^+^ regulatory T Cells suppress pathogenic Th17 cells and are impaired in multiple sclerosis. J. Immunol..

[83] Wang Y., Begum-Haque S., Telesford K.M., Ochoa-Reparaz J., Christy M., Kasper E.J., Kasper D.L., Robson S.C., Kasper L.H. (2014). A commensal bacterial product elicits and modulates migratory capacity of CD39^+^ CD4 T regulatory subsets in the suppression of neuroinflammation. Gut Microbes.

[84] Wang Y., Telesford K.M., Ochoa-Reparaz J., Haque-Begum S., Christy M., Kasper E.J., Wang L., Wu Y., Robson S.C., Kasper D.L. (2014). An intestinal commensal symbiosis factor controls neuroinflammation via TLR2-mediated CD39 signalling. Nat. Commun..

[85] Borsellino G., Kleinewietfeld M., Di Mitri D., Sternjak A., Diamantini A., Giometto R., Hopner S., Centonze D., Bernardi G., Dell’Acqua M.L. (2007). Expression of ectonucleotidase CD39 by Foxp3^+^ Treg cells: Hydrolysis of extracellular ATP and immune suppression. Blood.

[86] Peelen E., Damoiseaux J., Smolders J., Knippenberg S., Menheere P., Tervaert J.W., Hupperts R., Thewissen M. (2011). Th17 expansion in MS patients is counterbalanced by an expanded CD39^+^ regulatory T cell population during remission but not during relapse. J. NeuroImmunol..

[87] Tsutsui S., Schnermann J., Noorbakhsh F., Henry S., Yong V.W., Winston B.W., Warren K., Power C. (2004). A1 adenosine receptor upregulation and activation attenuates neuroinflammation and demyelination in a model of multiple sclerosis. J. Neurosci..

[88] Johnston J.B., Silva C., Gonzalez G., Holden J., Warren K.G., Metz L.M., Power C. (2001). Diminished adenosine A1 receptor expression on macrophages in brain and blood of patients with multiple sclerosis. Ann. Neurol..

[89] Mayne M., Shepel P.N., Jiang Y., Geiger J.D., Power C. (1999). Dysregulation of adenosine A1 receptor-mediated cytokine expression in peripheral blood mononuclear cells from multiple sclerosis patients. Ann. Neurol..

[90] Wan P., Liu X., Xiong Y., Ren Y., Chen J., Lu N., Guo Y., Bai A. (2016). Extracellular ATP mediates inflammatory responses in colitis via P2 x 7 receptor signaling. Sci. Rep..

[91] Flye M.W., Yu S. (1987). The synergistic effect of superoxide dismutase and adenosine triphosphate-MgCl_2_ on acute hepatic ischemia. Transplant. Proc..

[92] Ohana G., Cohen S., Rath-Wolfson L., Fishman P. (2016). A3 adenosine receptor agonist, CF102, protects against hepatic ischemia/reperfusion injury following partial hepatectomy. Mol. Med. Rep..

[93] Hart M.L., Gorzolla I.C., Schittenhelm J., Robson S.C., Eltzschig H.K. (2010). SP1-dependent induction of CD39 facilitates hepatic ischemic preconditioning. J. Immunol..

[94] Mediero A., Perez-Aso M., Cronstein B.N. (2013). Activation of adenosine A(2A) receptor reduces osteoclast formation via PKA- and ERK1/2-mediated suppression of NFkappaB nuclear translocation. Br. J. Pharmacol..

[95] Vincenzi F., Corciulo C., Targa M., Merighi S., Gessi S., Casetta I., Gentile M., Granieri E., Borea P.A., Varani K. (2013). Multiple sclerosis lymphocytes upregulate A2A adenosine receptors that are antiinflammatory when stimulated. Eur. J. Immunol..

[96] Varani K., Massara A., Vincenzi F., Tosi A., Padovan M., Trotta F., Borea P.A. (2009). Normalization of A2A and A3 adenosine receptor up-regulation in rheumatoid arthritis patients by treatment with anti-tumor necrosis factor alpha but not methotrexate. Arthritis Rheum..

[97] Vincenzi F., Padovan M., Targa M., Corciulo C., Giacuzzo S., Merighi S., Gessi S., Govoni M., Borea P.A., Varani K. (2013). A(2A) adenosine receptors are differentially modulated by pharmacological treatments in rheumatoid arthritis patients and their stimulation ameliorates adjuvant-induced arthritis in rats. PLoS ONE.

[98] Decking U.K., Schlieper G., Kroll K., Schrader J. (1997). Hypoxia-induced inhibition of adenosine kinase potentiates cardiac adenosine release. Circ. Res..

[99] Chouker A., Thiel M., Lukashev D., Ward J.M., Kaufmann I., Apasov S., Sitkovsky M.V., Ohta A. (2008). Critical role of hypoxia and A2A adenosine receptors in liver tissue-protecting physiological anti-inflammatory pathway. Mol. Med..

[100] Flogel U., Burghoff S., van Lent P.L., Temme S., Galbarz L., Ding Z., El-Tayeb A., Huels S., Bonner F., Borg N. (2012). Selective activation of adenosine A2A receptors on immune cells by a CD73-dependent prodrug suppresses joint inflammation in experimental rheumatoid arthritis. Sci. Transl. Med..

[101] El-Tayeb A., Michael S., Abdelrahman A., Behrenswerth A., Gollos S., Nieber K., Muller C.E. (2011). Development of Polar Adenosine A2A Receptor Agonists for Inflammatory Bowel Disease: Synergism with A2B Antagonists. ACS Med. Chem. Lett..

[102] Ochaion A., Bar-Yehuda S., Cohen S., Amital H., Jacobson K.A., Joshi B.V., Gao Z.G., Barer F., Patoka R., Del Valle L. (2008). The A3 adenosine receptor agonist CF502 inhibits the PI3K, PKB/Akt and NF-kappaB signaling pathway in synoviocytes from rheumatoid arthritis patients and in adjuvant-induced arthritis rats. Biochem. Pharmacol..

[103] Silverman M.H., Strand V., Markovits D., Nahir M., Reitblat T., Molad Y., Rosner I., Rozenbaum M., Mader R., Adawi M. (2008). Clinical evidence for utilization of the A3 adenosine receptor as a target to treat rheumatoid arthritis: Data from a phase II clinical trial. J. Rheumatol..

[104] Fishman P., Cohen S. (2016). The A3 adenosine receptor (A3AR): Therapeutic target and predictive biological marker in rheumatoid arthritis. Clin. Rheumatol..

[105] Bar-Yehuda S., Rath-Wolfson L., Del Valle L., Ochaion A., Cohen S., Patoka R., Zozulya G., Barer F., Atar E., Pina-Oviedo S. (2009). Induction of an antiinflammatory effect and prevention of cartilage damage in rat knee osteoarthritis by CF101 treatment. Arthritis Rheum..

[106] Guzman J., Yu J.G., Suntres Z., Bozarov A., Cooke H., Javed N., Auer H., Palatini J., Hassanain H.H., Cardounel A.J. (2006). ADOA3R as a therapeutic target in experimental colitis: Proof by validated high-density oligonucleotide microarray analysis. Inflamm. Bowel Dis..

